# Medical Dominance in Global Health Institutions as an Obstacle to Equity and Effectiveness: Comment on "Power Dynamics Among Health Professionals in Nigeria: A Case Study of the Global Fund Policy Process"

**DOI:** 10.34172/ijhpm.2022.7734

**Published:** 2023-01-04

**Authors:** Sarah L. Dalglish, Olutobi A. Sanuade, Stephanie M. Topp

**Affiliations:** ^1^Department of International Health, Johns Hopkins School of Public Health, Baltimore, MD, USA.; ^2^Institute for Global Health, University College London, London, UK.; ^3^Department of Population Health Sciences, Spencer Fox Eccles School of Medicine at the University of Utah, Salt Lake City, UT, USA.; ^4^College of Public Health Medical and Veterinary Sciences, James Cook University, Townsville, QLD, Australia.

**Keywords:** Nigeria, Power, Medical Professionals, Global Health, Decolonization

## Abstract

Medical professionals exercised structural and productive power in the Global Fund’s Country Coordinating Mechanism (CCM) in Nigeria, directly impacting the selection of approaches to HIV/AIDS care, as described in a case study by Lassa and colleagues. This research contributes to a robust scholarship on how biomedical power inhibits a holistic understanding of health and prevents the adoption of solutions that are socially grounded, multi-disciplinary, and co-created with communities. We highlight Lassa and colleagues’ findings demonstrating the ‘long arm’ of global health institutions in country-level health policy choices, and reflect on how medical dominance within global institutions serves as a tool of control in ways that pervert incentives and undermine equity and effectiveness. We call for increased research and advocacy to surface these conduits of power and begin to loosen their hold in the global health policy agenda.

*Medical doctors use medical language, which does not lead to a meaningful discussion with other occupations during meetings. So when I say [they] dominate, it is more about the type of language they use*.” Such are the words of one member of the Global Fund’s Country Coordinating Mechanism (CCM) in Nigeria, as reported by Lassa and colleagues in their case study of power dynamics in health policy-making.^1^ A key finding of their study was the dominance of medical professionals, specifically allopathic physicians, in decision-making spaces in Nigeria, who leveraged both structural power (using professional monopoly to enforce an occupational hierarchy) and productive power (using privileged access to a specialized knowledge base to frame the discourse on problems and solutions) to direct efforts and determine solutions for strengthening HIV/AIDS care.

 In health policy discussions, medical dominance occurs when allopathic medicine is positioned as the sole or primary framework for understanding and responding to health problems, with medical doctors correspondingly elevated as the most knowledgeable experts and decision-makers. Medicalized approaches to public health are reductionist, seek causes in biology rather than social or environmental factors, are individualistic rather than collectively minded, and focus narrowly on clinical and/or technological interventions.^2^ The medicalization of health issues from a macro (ie, policy or prioritization) perspective and the related question of medical dominance has been examined mainly in Western countries.^3^ However in recent years medicalized approaches to health have been increasingly understood as part of the colonial inheritance in many low- and middle-income countries.^4^ For example in Nigeria, where Lassa et al report on medical dominance in HIV/AIDS policy-making, the medical system continues to emphasize hospital-based curative care, benefiting the urban elite — rather than building a strong and equitable primary healthcare system that draws on multiple sectors to promote health and prevent illness amongst the whole population.^5^

 As such, Lassa and colleagues make a useful addition to a long tradition of public health and anthropological scholarship calling out biomedical power as detrimental to operationalizing health as a holistic, socially-embedded concept. But more work is needed to draw attention to how medical dominance prevails in the ‘high spheres’ of global health and how it perverts incentives, results in blinkered advice, and can harm rather than improve equity and effectiveness at every level. Global health institutions, including the World Health Organization (WHO), major multi-lateral bodies and global health initiatives, and bilateral and private donor agencies, have rarely questioned the dominance of medical professionals within their ranks and medical discourse in their strategies – nor the economic thinking and cost-effectiveness calculations that are used to further buttress this dominance. A medicalized framing is evident across a plethora of global health issues, and the goal-oriented structures of global health institutions, and competition between them, incentivize the application of biomedical solutions.^2^

 Medical dominance, exerted via structural and productive power, means that global health institutions rely on narrow conceptions of knowledge to guide their responses to health issues, often excluding or only superficially including lived experience, social policy expertise, and knowledge derived from non-positivist paradigms such as Indigenous methodologies, participatory action research, and even much of mainstream social science.^6^ These types of knowledge remain largely absent from the deliberative and decision-making processes of most major global health institutions – as does the practical wisdom (‘phronesis’) of how to implement interventions and policies.^7^ Dismissal of non-medical knowledge that could inform health strategies was evident in Lassa and colleagues’ study, where respondents said members of community-based organizations and patient groups did not have the ‘sophistication [of] MBBS medical doctors.’ As a guide to decision-making, the obsession with quantifying the impact of targeted, disease-specific, medical solutions – sometimes called the ‘Gates approach’ – is much criticized.^8,9^ Yet in global health spaces, this narrow, highly technical approach merely compounds the problems caused by the dominance of medicine, with its prioritization of quantifiable knowledge rendered ever-more ‘scientific’ by advances in machine-powered calculation.

 With such epistemological underpinnings, it should come as no surprise when so-called ‘solutions’ to complex and highly contextual health problems are, in effect, pre-determined, even in ‘country-led’ collaborations such as the Global Fund’s CCMs. “*It seems they have the answers to the questions they want you to answer*.” “*Their system is so rigid, everything is already spoon feeding*.” “*A path is shaped for you to follow.*” The words of the Nigeria CCM members interviewed by Lassa and colleagues indicate that a medicalized approach to HIV programming was in fact a non-choice – demonstrating how donor prerogatives drive funding allocations regardless of local priorities, drawing on the combined structural and productive power of global health institutions in the process.^10^ In this context, we can better understand the finding that Nigerian medical professionals sought to advance their own power and influence in health system decision-making by participating in these forums – and recommending medicalized solutions to public health problems.

 Despite the existence of a robust critical literature that situates healthcare as but one determinant of population health, the medical professionals who make up the leadership of many global health institutions, as well as in countries, are not equipped by their training to work in teams to address these determinants. As Naidu and Abimbola describe, Eurocentric medical – and we would argue public health – education as practiced around the world crowds out approaches to caring for people’s health that are more holistic, people-centered and equity-oriented, such as the Ife Philosophy of medical and health professionals education in Nigeria, which trained doctors as part of multi-disciplinary teams providing community-based primary healthcare, or the Aboriginal Community-Controlled Health Services in Australia.^4^ The oft-cited ‘barefoot doctors’ in China and other community health workers are frequently harkened back to in the global health discourse, in fond remembrance of Alma-Ata and continuing calls for more comprehensive notions of primary healthcare.^11^ In the most well-endowed global health initiatives, meanwhile, the focus on medicalized solutions continues largely undisturbed.

 Indeed, global health institutions today are arguably constitutionally incapable of producing policies and interventions that can realize the ambition of truly comprehensive primary healthcare. For instance, Lassa and colleagues described how in the Global Fund’s CCM in Nigeria, social interventions were de-emphasized in favor of biomedical content, so as to adhere to WHO guidelines and pass muster with the Global Fund’s Technical Review panel. Similarly, in Mozambique, rapid scale-up of technical HIV ‘care’ with financing from the World Bank, the Global Fund, the Clinton Foundation and President’s Emergency Plan for AIDS Relief was destructive to relationships between patients and caregivers, crowding out non-clinical forms of care, such as prayer and ‘motherly’ attention.^12^ In these cases and others, outreach to and partnership with people and communities, particularly marginalized ones, was subsumed into a medicalized framework that was not only exclusionary, but actively undermined critical forms of health ‘care.’

 The dominance of biomedical cadres, epistemologies and discourses in global health institutions limits the effectiveness of the interventions they propose, support and finance. In the case study of Nigeria’s CCM, Lassa and colleagues identified a strong emerging theme of ‘wasted antiretrovirals’ due to lack of uptake of the clinical HIV programming on offer, with over 20 tons of expired commodities left at central medical stores and 15 tons at state level stores, according to an audit report. The focus on purchasing commodities exemplifies how medicalization of health creates ‘too simplistic a view of making more modern medical treatments available to more people’ (Benatar, cited in Clark^2^), failing to recognize the intersecting social, economic and cultural conditions that must be in place to ensure a corresponding number of patients seek to use them. In the early 2010s, the Global Fund responded to significant criticism and pressure to shift its disease-focused and top-down approach to include health systems strengthening, yielding some improvements.^13^ But Lassa and colleagues, and earlier research,^14^ demonstrate how the structural influence of medical power in the broader global health environs continue to shape and narrow the focus of such initiatives.

 Medicalization can result in successful outcomes when viewed from certain angles. A recent evaluation of the Global Fund said the partnership had underperformed in building strong and resilient health systems due to its focus on disease-specific goals, while nonetheless touting the 44 million lives saved by the Fund since its inception.^13^ This framing gives the truth of the matter: despite sometimes aspiring to build durable health systems that serve populations including those traditionally excluded, global health initiatives remain fundamentally defined by, and focused on, activities that enable quantification of disease reduction and lives saved. Forty-four million lives is no small number. But it should not obscure the fact that the medicalization of health issues, via approaches that are focused on quantifiable technical or clinical interventions and designed without meaningful input from non-medical stakeholders, are also tightly linked to the ongoing colonial agenda of global health. Indeed, who are these numbers designed to appeal to? As scholars of Indian medical history have demonstrated, medicalization is not a regrettable outcome of historical contingency.^15^ Allopathic medicine is a tool in ongoing efforts by powerful states and actors to exert control in what should be a leading site of cooperation – the preservation and protection of people’s health.

 Lassa and colleagues’ research is a reminder that breaking the hold of medical dominance in global health institutions is necessary if we wish to make best use of limited resources to improve population health. Yet it will be a long row to hoe. Doing so will require a collective push from multiple directions, including research, civil society, and even political pressure to overcome deeply rooted power dynamics. Global health bodies and the academic institutions which are so tightly linked to them can start by meaningfully engaging in a learning agenda to finance, publish, collate and publicize research that demonstrates the pitfalls of medicalization and the ways in which holistic approaches are superior in terms of equity, justice, and basic effectiveness in promoting and protecting population health. Direct and robust advocacy is necessary to reveal and draw attention to the workings of power in global health institutions to challenge the ongoing narrative of disinterested (apolitical) investment in solving technical (medical) problems, and to surface conduits of power within the processes and policy agendas of such initiatives, and their impacts on the broader system. For their part, donor agencies will need to have faith and be patient. The most transformational development programs, which build institutions and encourage policy reform, are often those least likely to be precisely and easily measured.^8^

 Lassa and colleagues’ research identifies and names a power dynamic amongst a small group of actors that has had major consequences for HIV interventions in Nigeria. Following the trail of evidence leads straight to the biggest behemoths in global health.

## Ethical issues

 Not applicable.

## Competing interests

 Authors declare that they have no competing interests.

## Authors’ contributions

 SLD conceived of the commentary and wrote the first draft of the manuscript. OAS and SMT provided critical revisions for important intellectual content. SLD finalized the manuscript with the input and approval of OAS and SMT.
